# Cardiovascular health of offspring conceived by assisted reproduction technology: a comprehensive review

**DOI:** 10.3389/fcvm.2024.1287060

**Published:** 2024-01-16

**Authors:** Jie Li, Yang Liu, Hefeng Huang, Li Jin

**Affiliations:** Obstetrics and Gynecology Hospital, Institute of Reproduction and Development, Fudan University, Shanghai, China

**Keywords:** cardiovascular diseases, assisted reproductive technology (ART), epigenetics, embryo development, DNA methylation

## Abstract

Recently, the use of assisted reproductive technology (ART) has rapidly increased. As a result, an increasing number of people are concerned about the safety of offspring produced through ART. Moreover, emerging evidence suggests an increased risk of cardiovascular disease (CVD) in offspring conceived using ART. In this review, we discuss the epigenetic mechanisms involved in altered DNA methylation, histone modification, and microRNA expression, as well as imprinting disorders. We also summarize studies on cardiovascular changes and other risk factors for cardiovascular disease, such as adverse intrauterine environments, perinatal complications, and altered metabolism following assisted reproductive technology (ART). Finally, we emphasize the epigenetic mechanisms underlying the increased risk of CVD in offspring conceived through ART, which could contribute to the early diagnosis and prevention of CVD in the ART population.

## 1 Introduction

Assisted reproductive technology (ART) encompasses a range of procedures, including *in vitro* fertilization (IVF), intracytoplasmic sperm injection (ICSI), oocyte donation (OD), superovulation, freeze-thawing, and other related techniques. The use of ART has increased in recent years owing to the increasing incidence of infertility in the population. In the United States, approximately 1.9% children are conceived through ART (1); similarly, over 1.0% of all births result from ART in mainland China (2). Moreover, it is predicted that over 150 million children, or 1.4% of the global population, will be conceived with ART by the end of the century (3).

It is widely accepted that the risk of developing diseases is linked to critical developmental periods such as the periconceptional, prenatal, and early postnatal stages (4). Barker's Developmental Origins of Health and Disease (DOHaD) theory suggests that changes in the conception microenvironment during both the intrauterine and postnatal periods can result in long-term damage, particularly in the form of cardiovascular and metabolic diseases (5, 6). Recently, adverse environmental exposure of oocytes during the pre-gestational period has shown to have lasting effects on offspring (7). Thus, concerns have been raised that ART techniques may interfere with early development and lead to long-term disorders in offspring.

The primary reason for premature death in China is due to cardiovascular disease (CVD) (8, 9). Additionally, in the Western, there has been a gradual rise in the frequency and prevalence of cardiovascular diseases, whether they are congenital or acquired, which has contributed to changes in risk factor profiles among children and young adults (10). Thus, CVD in offspring conceived via ART has received considerable attention, and emerging evidence suggests congenital heart defects (CHD) and an increased cardiovascular risk in offspring conceived using ART (11–21). In this comprehensive review, we collected evidence from humans and animals to explore the epigenetic alterations induced by ART and their subtle consequences on offspring cardiovascular health.

## 2 Epigenetic modification and ART-induced cardiovascular dysfunction

To ensure the quality of embryos and pregnancy outcomes, gametes are protected from environmental stress in the female reproductive tract by the tubal fluid during the pre-fertilization period *in vivo*. Despite efforts to minimize *in vivo* environmental stimuli during ART procedures, they still differ from those in the female reproductive tract. IVF and ICSI are two common ART used to help couples conceive. The process of IVF involves stimulating the ovaries to produce multiple eggs, retrieving the eggs, fertilizing them with sperm in a laboratory dish, and then transferring the resulting embryos into the uterus. ICSI is a type of IVF that involves injecting a single sperm directly into an egg to facilitate fertilization. Both procedures offer hope to couples struggling with infertility. More specifically, the ART setting involves exposure to a range of stimuli, including superovulation (22), cryopreservation (23, 24), exposure to various types of lights (25), fluctuations in pH and temperature (26, 27), changes in oxygen tension (28), culture media that contain specific substances such as Fe2+ and Cu2+ (29), and gamete or embryo manipulation. All these ART interventions act during gamete-imprinted gene reprogramming and embryonic gene demethylation (30). Thus, with exquisite sensitivity to environmental insults, the trajectories of gametic and embryonic development can be easily affected.

Epigenetics is the study of changes in gene expression that occur without alterations to the underlying DNA sequence. Epigenetic modifications can influence gene expression by modifying chromatin structure and DNA accessibility. These modifications can be influenced by environmental factors and can have long-lasting effects on an organism's phenotype (31). The relationship between epigenetics, cardiac development, and disease has been supported by a growing body of evidence (32). Epigenetic markers, such as DNA methylation and histone modification, are established in germ cells and maintained throughout embryonic development and postnatal life (33). Controlled ovarian stimulation acts during the period when the imprinted genes of oocytes are reprogrammed, while *in vitro* embryo culture acts during the sensitive period of gene demethylation (30). Consequently, ART can cause epigenetic dysregulation in embryos and adult offspring, ultimately affecting cardiovascular health.

### 2.1 DNA methylation

DNA methylation is a type of epigenetic modification that involves the addition of methyl groups to cytosine or adenine bases in DNA. This process primarily occurs in CpG (5′-C-phosphate-G-3′) dinucleotides (34). Non-CpG methylation is found in embryonic stem cells and non-dividing cells, such as neurons, and plays a role in regulating cell type-specific functions (35). CpG sites are highly concentrated in genomic regions called CpG islands, which are mainly located in promoter regions and are typically unmethylated. CpG sites outside of these islands are often methylated in mammals (36). DNMT3A and DNMT3B (DNA methyltransferase 3A and 3B) are responsible for *de novo* DNA methylation (37).

#### 2.1.1 ROS and its role in the DNA methylation process

**Figure 1:**
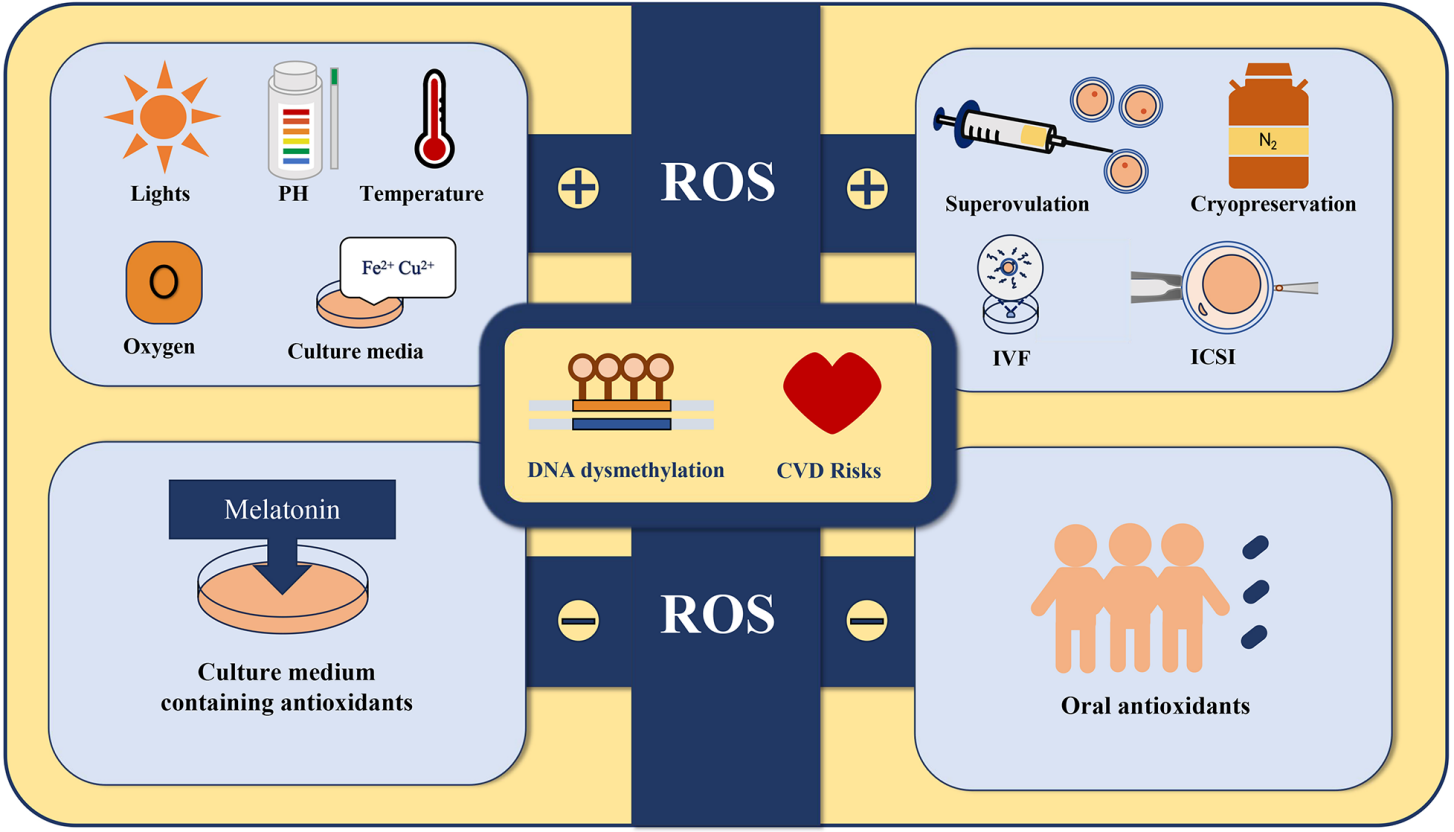
All the external stimuli including superovulation, cryopreservation (23, 24), exposure to various types of lights, fluctuations in pH and temperature, changes in oxygen tension, culture media that contain specific substances such as Fe2+ and Cu2+, and gamete or embryo manipulation may induce high ROS production in the ART setup. Adding antioxidants such as melatonin to the culture medium and administering antioxidant supplements to the offspring may protect their cardiovascular health by suppressing ROS production.

Oxidative stress (OS) is associated with excess reactive oxygen species (ROS) and a decrease in antioxidant enzymes (38). To ensure the quality of embryos and the outcome of pregnancy, gametes in the female reproductive tract are protected from environmental stress by tubal fluid during the pre-fertilization period. After shedding cumulus cells, the embryo depends on tubal fluid and internal antioxidant activities to gain protection against ROS-induced stress. All the external stimuli mentioned above may induce high ROS production in the ART setup (Figure 1). Excess ROS has been proposed to cause severe damage during embryonic development, and increasing evidence shows that the production of ROS is important for the development of the heart and the pathogenesis of cardiovascular disease (39, 40).

A theory of ROS induction has recently been proposed to explain the mechanisms underlying the establishment of DNA hypermethylation or hypomethylation (41–43). Elevated levels of ROS and DNA methylation have been observed in various types of cancer cells (43). ROS attack-induced hydroxylation of methylcytidine generates 5hmC, which disrupts the accurate transmission of genomic methylation patterns (44). ROS facilitate the hypermethylation of NDRG2 promoters in a manner that is dependent on DNMTs, key enzymes in DNA methylation (45). Moreover, ROS can induce site-specific hypermethylation by upregulating DNMTs or forming new DNMT-containing complexes (43). However, as ROS can affect numerous cellular processes, alternative mechanisms may contribute to DNA methylome alterations (46).

#### 2.1.2 The relationship between imprinting abnormalities in ART offspring and CHD

Genomic imprinting is an epigenetic process that affects a specific set of mammalian genes, resulting in a monoallelic expression pattern that is inherited from one parent. To distinguish between parental alleles, imprinted genes are marked epigenetically in gametes at imprinting control elements using DNA methylation at the very least (47).

According to a recent study, ART causes abnormal expression of 1,060 genes in the mouse heart. The genes identified are mainly associated with RNA synthesis, processing, and the development of the cardiovascular system. The core interacting factors include Ccl2, Ptgs2, Rock1, Mapk14, Agt, and Wnt5a. Further investigation revealed that 42 epigenetic modifiers were abnormally expressed in the heart. In addition, in the hearts of ART offspring, the expression of imprinted genes Dhcr7, Igf2, Mest, and Smoc1 was found to be reduced, whereas the DNA methylation levels of the Igf2- and Mest-imprinting control regions (ICRs) were abnormally increased (19). Superovulation in adult mice has also been linked to alterations in Sgce and Zfp777 imprinted genes, whereas *in vitro* culture of follicles from the early pre-antral stage resulted in globally reduced methylation and heightened variability at imprinted loci in blastocysts (48).

Additionally, non-stimulated oocytes had lower methylation percentages in the imprinted genes APEG3, MEG3, and MEG9 and were higher in TSSC4 compared to stimulated oocytes in a bovine model. In terms of the CGI of imprinted genes, non-stimulated oocytes had higher methylation percentages of MEST (PEG1), IGF2R, GNAS (SCG6), KvDMR1, ICR, UMD, and IGF2. In another region around IGF2, non-stimulated oocytes had lower methylation percentages than stimulated oocytes (22).

Imprinting aberrations in SNRPN are involved in the pathogenesis of CHD (49). A study that followed children over time found who were conceived through ICSI had a higher incidence of SNRPN DMR hypermethylation. This occurrence remained stable even after the children reached seven years of age, indicating that these changes may persist over time (50). In addition to humans, oocyte vitrification can also result in the loss of Snrpn DNA methylation in mouse blastocysts. This loss of methylation is primarily caused by a reduction in DNMTs after oocyte vitrification (51).

It was reported that the skewed sex ratio is a result of developmental defects during the peri-implantation stage, which predominantly affect females. An imbalanced sex ratio, which serves as an indicator of reproductive hazards, has been reported in mouse (52), bovine (53), porcine (54) embryos as well as in newborns from human IVF procedures (55, 56). Male bias has been observed in both mammals and humans (52, 56). Given that women have a more favorable cardiovascular risk profile than men, particularly at younger ages (57), this sex bias may contribute to the increasing CVD risk following ART. Furthermore, these defects were reported to be caused by impaired imprinted X chromosome inactivation (iXCI) due to reduced expression of Rnf12/Xist in mice (52). Although humans do not undergo imprinted XCI like mice, they still exhibit a male bias in IVF. This suggests that impaired X chromosome inactivation may occur due to reasons other than imprinting in humans.

Although there is evidence of an increase in imprinting disorders in children conceived through IVF and ICSI, there is currently insufficient evidence to establish a link between ART and the methylation of other imprinted genes. Further controlled studies using standardized methodologies in larger and more clinically defined populations are required to better understand the relationship between ART and the methylation of imprinted genes.

#### 2.1.3 eNOS and Ang II: potential therapeutic targets

Vasculature from ART mice displayed endothelial dysfunction and increased stiffness, which could lead to arterial hypertension *in vivo*. The underlying mechanism was shown to be the decreased expression and function of endothelial eNOS, which is an isoform of nitric oxide synthase that is specific to endothelial cells (14). eNOS in endothelial cells (ECs) produces nitric oxide (NO), which contribute to the maintenance of proper vascular tone and systemic hemodynamics (58). Hypermethylation of the eNOS promoter can lead to decreased expression and function of endothelial eNOS, which can cause endothelial dysfunction and increased vascular stiffness in ART mice. This could lead to arterial hypertension *in vivo* (14). Moreover, addition of melatonin to culture media prevents the ART-induced eNOS dysmethylation and normalizes vascular dysfunction (59). Melatonin is known for its strong antioxidant activity, which helps scavenge free radicals, such as ROS (60). Using a culture medium containing antioxidants or vitrification and warming solutions supplemented with antioxidants has been shown to have a significant positive impact on the *in vitro* development of mouse preimplantation embryos, as well as on subsequent fetal development post-transfer (59, 61–63). In children with ART, the short-term use of oral antioxidants has also been found to have beneficial effects on vascular function (64).

The renin-angiotensin system (RAS) is a crucial physiological system that is responsible for regulating blood pressure and ensuring proper fluid and electrolyte balance within the body. In this system, angiotensin II (Ang II) is the primary effector hormone that acts as a systemic hormone and locally produced factor (65). Recent research discovered that ART-conceived mice showed an increased expression of myocardial AT1R, which encodes the type 1 receptor of Ang II, beginning at three weeks after birth. This increase was confirmed at 10 weeks and 1.5 years of age compared to the non-IVF group (66). Another study found that the vasoconstrictor response to ANG II was significantly higher in ART mice than that in non-ART mice. This increased response was associated with increased AT1R expression. The study also found that hypomethylation of two CpG sites located in the At1bR promoter led to increased gene transcription, further contributing to exaggerated vasoconstrictor responsiveness in ART mice (67). In human, a previous study reported that the IVF-ET group showed decreased mRNA expression of DNMT3A in the umbilical vein and hypomethylation of the AGTR1 gene, which codes for AT1R. These results indicate that IVF-ET treatments can alter Ang II-mediated vasoconstriction in umbilical veins, possibly due to increased expression of AT1R caused by dysmethylation (68).

### 2.2 Other epigenetic alternations in ART offspring

#### 2.2.1 Histone modification

In addition to DNA methylation, histone modification is another crucial epigenetic mechanism. Trimethylation of lysine 4 (K4) and 27 (K27) of histone H3 (H3K4me3 and H3K27me3, respectively) is associated with gene activation and repression, respectively (69). Research studies have shown that histone lysine methylation plays a crucial role as an epigenetic regulator of heart development. Abnormalities during this process can lead to cardiac anomalies (32). According to the study by Maldonado et al. (70), the levels of H3K4me3 were approximately 20% lower, while the levels of H3K27me3 were higher in frozen-thawed bovine blastocysts compared to fresh embryos. Based on this hypothesis, the observed phenomenon was thought to be caused by cellular stress, particularly oxidative stress. To test this hypothesis, the blastocysts were placed under either normoxic (5%) or hyperoxic (20%) conditions and it was demonstrated that the levels of H3K4me2 and H3K9me2 were altered. Another study showed that the histone and chromatin status of mouse embryos was altered by *in vitro* culture rather than by prior vitrification and warming (71).

#### 2.2.2 Micro RNA

Micro RNA (miRNA) are small non-coding RNA molecules approximately 22 bp in length. It can bind to the three prime untranslated region (3’UTR) of target mRNAs, causing cleavage or translational repression (72). They play a significant role in cardiac muscle proliferation and differentiation (73).

Dysregulated miRNA expression profiles have been observed in IVF-TE human placental tissues (74) and mouse embryos (75). Elevated levels of miR-100, miR-297, and miR-758 have been observed in the myocardial tissue of mice conceived through IVF compared to naturally bred mice of the same age (66), which might contribute to cardiovascular malformations by RAS. In addition, miR-1 (76), miR-206 (76) and miR-421 (77) might contribute to the pathology of tetralogy of Fallot (TOF), the most common type of cyanotic CHD. However, owing to limited research, the association between altered miRNAs in IVF offspring and CVD risk remains unclear.

## 3 Cardiovascular alterations induced by assisted reproductive technology

### 3.1 Alterations in cardiac structure and function

Cardiac remodelling and dysfunction have been reported in ART compared to spontaneously conceived fetuses (17, 78), which could persist in infants, children, and adolescents (15, 79, 80). A recent study found lower LV systolic function in ART subjects compared to spontaneously conceived peers. However, after adjusting for birth weight percentiles and gestational age, M-mode-assessed LV systolic function showed no significant differences between the groups (21). Similarly, lower diastolic function in ART subjects was found in another study after adjusting for age, birth weight percentile, and gestational age (81), and there were no significant differences in LV diastolic function between the two groups.

CHD is the most prevalent type of birth defect and is characterized by congenital malformations of the heart walls, valves, and blood vessels (82). A large retrospective cohort study consisting of 507,390 patients reported a significant association between ART and CHD in general, without specifying the subtype. However, after assessing the mediation of twin pregnancies (which accounted for 87% of the total), the same correlation was found to be statistically insignificant (83). In a 2018 cohort study, an increased incidence of CHD (1.8% vs. 1%) was reported without specifying the subtype. Upon further analysis, it was found that the incidence of nonsevere CHD was higher in children conceived through ART (2.2%) compared to those conceived naturally (1%). However, when considering severe CHD, the incidence was comparable between ART and NC (1.4% and 1.2%, respectively) (84). Recently, a systematic review extracted twenty-four studies on the incidence of CHD in ART was conducted between January 2011 and May 2022 (20). It concluded that the incidence of CHDs among offspring conceived by IVF was 3% and decreased to 1% for major CHDs only. Compared to non-ART pregnancies, there appears to be an increased risk of CHDs in ART pregnancies, particularly minor cases that do not require surgical correction. However, evidence is insufficient to determine the actual risk of developing major CHDs. Additionally, certain confounding factors such as maternal age and male infertility may play a critical role in determining the increased risk of CHDs (85–87).

Owing to the conflicting results among studies, further research is needed to validate the evidence and accurately determine the risk of alterations in cardiac structure and function following ART pregnancies. More extensive research with larger sample sizes and extended follow-up periods is warranted.

### 3.2 Abnormal blood pressure

Increased arterial blood pressure was found in ART-conceived offspring (11, 88) compared to the control group. As mentioned previously, this could be a consequence of vascular dysfunction (88). Pulmonary hypertension in ART-conceived children has been reported under hypoxic and normoxic stress (13, 79, 89, 90), which may be attributed to decreased pulmonary vascular distensibility (89). A meta-analysis of over 35,000 mostly child offspring found that ART (compared to natural conception) resulted in similar blood pressure, heart rate, and glucose levels but higher cholesterol levels. Additionally, a long-term follow-up of 17,244 births (244 of which were ART) showed that children conceived through ART tended to have lower predicted systolic (SBP) and diastolic blood pressure (DBP) during childhood. However, as they entered young adulthood, there were slight indications of higher SBP and triglyceride levels. It's worth noting that most of these differences were not statistically significant (91).

Ovarian hyperstimulation syndrome (OHSS) is a significant and potentially dangerous complication of IVF. It is characterized by increased levels of estradiol in the bloodstream, enlargement of the ovaries with cysts, and a shift of fluid from the blood vessels to other areas of the body (92). According to a prior investigation, controlled ovarian hyperstimulation during IVF results in offspring with significantly higher SBP than those born through modified natural cycles (93). Accordingly, another study revealed that the systolic blood pressure SBP and DBP in children aged 3–6 years, who were conceived naturally, were significantly lower compared to the OHSS-ET group (94).

According to a meta-analysis conducted in 2017, a total of 19 studies were reviewed to better understand the health outcomes of children born through IVF-ICSI compared to those conceived naturally. These studies included 2,112 IVF-ICSI offspring and 4,096 naturally conceived offspring across various age groups from childhood to early adulthood. This study revealed that IVF-ICSI offspring had notably higher blood pressure levels than naturally conceived offspring. The weighted mean differences and confidence intervals were 1.88 mmHg [95% CI: 0.27, 3.49] for SBP and 1.51 mmHg [95% CI: 0.33, 2.70] for DBP, indicating a significant difference between the two groups (95).

Five animal studies reported varied results on the SBP/DBP and the mean blood pressures of the children at a specific time point between 9 and 52 weeks of age (14, 59, 67, 96, 97). Three studies conducted on 12–14-week-old male mice using telemetry (14, 59, 67) found that the mice conceived via IVF exhibited significantly higher mean fixed and continuous arterial pressures. Additionally, a study reported that female mice conceived through IVM had increased SBP levels at 1.5 years of age, but no significant increase was observed in male mice or those conceived through IVF or ICSI (97). More importantly, long-term exposure to high blood pressure can lead to structural abnormalities (98). Thus, differences in blood pressure are clinically important for early intervention of cardiac structure alterations in ART offspring.

### 3.3 Vascular dysfunction

A research team from Switzerland. paid significant attention to cardiovascular risks among ART offspring, both in humans (13, 88) and mice (14, 59). After assessing markers of early atherosclerosis in children (mean age, 11 years) who were conceived naturally and by ART (13), they found that carotid-femoral pulse-wave velocity (PWV), a proxy for elastic artery stiffness, was significantly faster in children who were conceived by ART than in control children. Defective flow-mediated dilation (FMD) of the brachial artery, which is related to endothelial dysfunction (99), and greater carotid intima-media thickness (cIMT) have also been found in children from ART. At the 5-year follow-up, the team reassessed the vascular function in these children (88). The alterations in FMD of the brachial artery, PWV, and cIMT were not only found to be persistent, but may also have the potential to translate into arterial hypertension, which has been demonstrated in several studies in humans (11, 15, 88). Furthermore, greater cIMT was confirmed in a smaller cohort (100). In addition to humans, ART mice exhibit signs of endothelial dysfunction and increased stiffness (14, 59, 101), which can even be transmitted to their offspring by male ART mice (14).

Conversely, a recent study in humans showed no differences in early markers of atherosclerosis (cIMT and arterial stiffness) between ART and non-ART groups at ages 22–35 years (102, 103). The study focused on individuals in early adulthood and used noninvasive techniques to identify early markers of subclinical atherosclerosis. However, the researchers did not examine the links between these markers and clinical cardiovascular events. Another study also found no significant difference in vascular function between children, adolescents, and young adults conceived through assisted reproductive technology and their spontaneously conceived peers (104). However, given the limited sample size in this study (66 ART and 86 naturally conceived offspring), larger multi-center studies are necessary to gather clinical evidence.

Collectively, ART may interfere with early development and lead to premature vascular dysfunction in the offspring, potentially due to the variety of *in vitro* manipulations and cultures from gametes to embryos involved in the technology. However, the relationship between these techniques and early atherosclerotic disorders has not been clearly defined and requires further investigation.

### 3.4 Cardiovascular risk factors in ART populations

#### 3.4.1 Perinatal complications

ART is often associated with adverse pregnancy outcomes, including fetal growth restriction, low birth weight, and preterm birth (PTB) (105), all of which are associated with higher CVD rates in adulthood (106–108). ART is also associated with an increased incidence of preeclampsia in mothers (109–111), which could have a negative effect on systemic and pulmonary vascular function of the offspring (12).

#### 3.4.2 Altered metabolism in ART offspring

Metabolic syndromes in the progression of CVDs, such as obesity, diabetes, dyslipidemia, and impaired glucose metabolism, are known cardiovascular risk factors (112). ART offspring show altered glucose homeostasis and exacerbated obesity in mice and humans (97, 113). In a recent study, ART offspring in childhood were found having similar TG levels, but higher TC (HDLc and LDLc) levels compared to NC; however, these differences were not observed in young adulthood. In contrast, as age increased, those conceived by ART had higher TG and lower HDLc levels than NC, although the differences were small and not statistically significant by age 26 (114). Higher TG levels are known to increase future cardiovascular disease risk, which might suggest an increased risk in ART-conceived offspring (115). Moreover, elevated maternal estrogen levels after ovulation induction have been linked to higher total cholesterol and low-density lipoprotein cholesterol levels in newborns (116). In mice, ICSI or *in vitro* oocyte maturation (IVM) has a significant impact on the hepatic expression and methylation of INSIG-SCAP-SREBP from young to old age (97), and these alterations are involved in cardiometabolic changes (117, 118). Collectively, these metabolic disorders may serve as early indicators of CVD risk (106, 119).

#### 3.4.3 Adverse intrauterine environment

The significance of the intrauterine environment cannot be overstated, as it serves as a mediator of the environment during vital developmental periods. Numerous studies have shown that an inadequate maternal diet (120), stress (121), and hormonal imbalances during pregnancy (122, 123) can affect the developmental programming of future generations. Women who undergo ART may face various challenges, including endocrine issues, advanced maternal age, chronic pelvic inflammation, and insulin resistance (124), all of which could contribute to cardiovascular dysfunction in the offspring. In addition, increased susceptibility to perinatal complications such as low birth weight and PTB during ART pregnancy may be partly due to a compromised intrauterine environment (95).

## 4 Conclusion and future perspectives

Although many studies have indicated a correlation between ART and an increased risk of CVDs among offspring, there is still conflicting evidence and no clear conclusion has been reached. Epigenetic alterations in ART populations have been reported in multiple studies, but the underlying mechanisms remain unclear. ROS could be one of the culprits for the high ROS production induced by external stimuli in the ART setup, and adding antioxidants to the culture media could mitigate the damage. Dysmethylation of AGTR1 and eNOS are shown to be associated with abnormal blood pressure levels. By applying the ROS inhibitor melatonin, the hypermethylation of eNOS can be effectively prevented. However, considering that ROS can affect numerous cellular processes, alternative mechanisms may contribute to epigenetic alterations. Moreover, considering the multifactorial nature of CVDs, adverse intrauterine environments, perinatal complications, and altered metabolism may play roles in the development of CVDs in ART offspring. Further research with larger, well-defined clinical ART populations and standardized methodologies is required, and more epigenetic experiments are needed to elucidate the potential mechanism of epigenetic inheritance in cardiovascular alterations in ART offspring. Overall, more attention should be paid to the CVD risks in offspring conceived through ART, which could not only contribute to the early diagnosis and prevention of CVD but also improve the safety and precision of ART.
